# Focal Causal Temporal Convolutional Neural Networks: Advancing IIoT Security with Efficient Detection of Rare Cyber-Attacks

**DOI:** 10.3390/s24196335

**Published:** 2024-09-30

**Authors:** Meysam Miryahyaei, Mehdi Fartash, Javad Akbari Torkestani

**Affiliations:** Department of Computer Engineering, Arak Branch, Islamic Azad University, Arak 38361-1-9131, Iran; m.miryahyaei@iau.ir (M.M.); ja.akbari@iau.ac.ir (J.A.T.)

**Keywords:** IoT security, focal causal temporal CNNs (FCTCNNs), imbalanced data problem, deep learning, intrusion detection system (IDS)

## Abstract

The Industrial Internet of Things (IIoT) deals with vast amounts of data that must be safeguarded against tampering or theft. Identifying rare attacks and addressing data imbalances pose significant challenges in the detection of IIoT cyberattacks. Innovative detection methods are important for effective cybersecurity threat mitigation. While many studies employ resampling methods to tackle these issues, they often face drawbacks such as the use of artificially generated data and increased data volume, which limit their effectiveness. In this paper, we introduce a cutting-edge deep binary neural network known as the focal causal temporal convolutional neural network to address imbalanced data when detecting rare attacks in IIoT. The model addresses imbalanced data challenges by transforming the attack detection into a binary classification task, giving priority to minority attacks through a descending order strategy in the tree-like structure. This approach substantially reduces computational complexity, surpassing existing methods in managing imbalanced data challenges in rare attack detection for IoT security. Evaluation of various datasets, including UNSW-NB15, CICIDS-2017, BoT-IoT, NBaIoT-2018, and TON-IIOT, reveals an accuracy of over 99%, demonstrating the effectiveness of FCTCNNs in detecting attacks and handling imbalanced IoT data with efficiency.

## 1. Introduction

The widespread adoption of the Internet of Things (IoT) has significantly impacted various domains, including smart cities, cameras, smart industrial equipment, and other aspects of daily life. On the other hand, the IoT environment deals with massive amounts of data that need to be protected from tampering or theft. Detecting security attacks against the IoT domain requires intelligent techniques rather than relying on signature matching. Machine learning (ML) and deep learning (DL) approaches are effective in identifying these attacks and predicting intrusion behavior based on unknown patterns [1,2,3]. Machine learning plays a crucial role in building network intrusion detection systems. However, machine learning models trained on imbalanced cybersecurity data cannot effectively detect minority data and, consequently, attacks [4,5].

Using imbalanced data in machine learning leads models to successfully predict normal data (majority class) but fail to detect minority and rare attacks. In such cases, the models assign rare data to the normal or majority data class [6,7]. This challenge becomes more problematic in multi-class classification, as accurately detecting minority attack types is more critical than identifying normal traffic [8,9]. One of the existing solutions to address and manage this challenge is the generation of synthetic data, achieved through methods like oversampling [10,11,12] and undersampling [13], to balance the data [14,15,16,17,18].

Studies focusing on this challenge often utilize methods such as the SMOTE family of algorithms [19,20,21], Generative Adversarial Networks (GANs) [22], and similar techniques. These methods primarily aim to produce synthetic data to augment the minority class [17,23]. In [24], the Bee Colony and Borderline SMOTE were used to address data imbalances for attack detection. In [5,25], ADASYN was employed for oversampling rare attacks. GANs were utilized in [4,22,23,26,27]; these methods have been somewhat more successful compared to SMOTE-based models. However, the quality of generated synthetic data and increased computational complexity due to the generation of new data are major limitations of such methods, leading to their partial success. Network traffic data are inherently classified as big data, and generating data in highly rare attack classes, which significantly differ from the majority class, results in a substantial increase in data volume and consequently in computational and temporal complexity.

In this paper, to overcome the data imbalance problem in attack detection within IoT, unlike existing methods, we propose a deep focal architecture based on causal temporal convolutional networks (TCNNs) that effectively manages data imbalances without data manipulation. This model transforms the multi-class classification problem into a binary classification and considers the population of each class for classification. In this model, a novel approach to addressing the data imbalance issue is proposed, which, unlike existing data-centric methods, is model-centric. This method considers and classifies both classes with nearly equal sample sizes at each level of its hierarchical structure. As a result, the model does not encounter data imbalance problems at any level of the proposed hierarchical structure. Consequently, the issue of data imbalance is managed without data manipulation or the generation of new synthetic data. The main components of our method are as follows:

1. Proposing a new binary deep model based on causal temporal convolutional networks (TCNN) for detecting rare attacks in traffic data streams.
2. Managing the data imbalance challenge structurally, without altering the data.
3. Transforming the multi-class attack detection problem into a binary classification problem and detecting two balanced types of attacks at each level of the deep hierarchical structure to address the challenge of data imbalance.
4. Utilizing a focal loss function in the proposed deep learning architecture.

Subsequently, in Section 2, we review the research background, including related studies. Section 3 introduces the proposed method, while Section 4 discusses the results obtained, and the Section 5 provides conclusions and future work directions.

## 2. Literature Review

### 2.1. Temporal Convolutional Neural Networks (TCNNs)

Temporal convolutional neural networks (TCNNs) are widely used in image recognition due to their powerful pattern recognition capabilities. Recently, their application has expanded to sequence data prediction, such as weather forecasts, stock markets, and more. Similar to a simple neural network, deep CNNs consist of multiple neurons connected through a hierarchical structure, and the weights between layers are trainable. Unlike other deep network structures, such as fully connected deep networks like Deep Belief Networks (DBN) and Sparse Autoencoder (SAE), CNNs can share weights between neurons of each layer, significantly reducing the network’s weight and preventing it from falling into local optima [28].

A convolutional neural network consists of an input layer, an output layer, and several hidden layers [29]. These layers are generally categorized into three main types: convolution layers, pooling layers, and fully connected layers. A convolutional neural network comprises a wide spectrum of convolution and pooling layers, followed by fully connected layers, meaning one or more fully connected layers are inserted after numerous convolution and pooling layers in the network.

**Figure 1:**
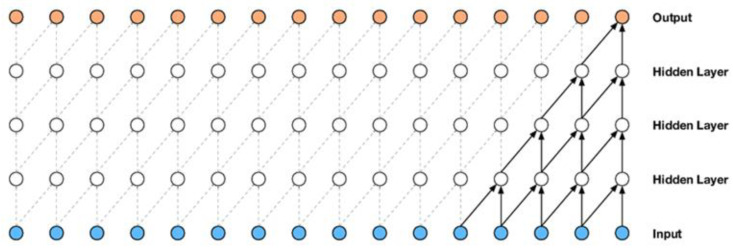
A stack of convolutional layers [32].

The main unit of a convolutional network is the convolution layer, where most computations happen. This layer comprises an array of ordered neurons, and its parameters consist of a set of learnable filters or kernels. These filters combine with the input data to produce a 2D feature map, which, when stacked along the depth dimension, generates the layer’s output. Neurons in this layer share weights, thus reducing the network’s complexity by keeping the number of parameters low [30]. The convolution layer also comes in other forms, such as the causal convolution layer, which is used in temporal data. Causal convolution represents a specific convolution layer that guarantees the model’s adherence to the sequential order in which the data are processed [31]. Figure 1 presents a stack of causal convolution layers.

The primary structure of a convolutional network is composed of a sequence of convolution layers and pooling layers. The role of the pooling layer is to decrease the spatial dimensions of the feature maps generated by the convolution layer while keeping important information. By doing so, the pooling layer reduces the network’s parameter count, resulting in a reduction in the overall computational intricacy. This layer effectively controls the problem of overfitting. Some common pooling operations include max pooling, average pooling, r Stochastic Pooling [33], spectral pooling [34], spatial pyramid pooling, and multiscale orderless pooling [30,35].

Neurons in the fully connected layer are fully connected to all neurons in the previous layer, resembling a standard neural network. High-level reasoning occurs in this layer. Neurons are disordered spatially and are one-dimensional, meaning there cannot be a fully connected layer immediately following another fully connected layer. Lately, in certain architectural designs, the global average pooling layer has been introduced as a substitution for the fully connected layer [30].

### 2.2. The Cost-Sensitive Loss Function in Deep Neural Network Learning

A deep neural network uses a loss function to optimize its parameters. Typically, the default loss function is cross-entropy, using the sigmoid function for binary class classification and the SoftMax function for multi-class classification. The regular cross-entropy loss used for classification is mathematically defined as Equation (1):

(1) $$CE\left(p,y\right)=\left\{\begin{array}{c} -\log\left(P\right)\;\;\;\;if\;\;\;\;\;\;\;\;\;\;\;\;y=1\\ -\log\left(1-p\right)\;\;otherwise \end{array}\right.$$

The function $CE\left(p,y\right)$ represents the cross-entropy loss function, which is commonly used in classification tasks. Here is a detailed explanation of its parameters:

- *p*: The predicted probability that the instance belongs to the positive class (class 1).
- *y*: The true label, which can be 0 or 1.

When the true label y=1, the loss function becomes $-\log\left(P\right)$. This penalizes the model more if p is close to 0, which indicates a wrong prediction with high confidence.

When the true label y=0, the loss function becomes $-\log\left(1-p\right).$ This penalizes the model more if p is close to 1, indicating a wrong prediction with high confidence.

In an imbalanced dataset, a large class dominates the cost of a gradient. If $CE\;(p_{t})=CE\;(p,y)$, $\alpha_{t}$ balances the importance of positive and negative examples. Lin et al. [36] changed the cross-entropy error by adding a modulating factor (1 – *pt*)*γ* with an adjustable parameter γ ≥ 0, referred to as a focal loss function. Formally, the focal loss function is expressed as Equation (2), where γ represents a positive scaling factor:

(2) $$FL\left(p_{t}\right)=-\alpha_{t}{\left(1-p_{t}\right)}^{\gamma }\log\left(p_{t}\right)\;\;\;\;where\;\;\alpha_{t}=\left\{\begin{array}{c} \alpha \;\;\;\;\;\;\;\;\;if\;\;\;\;\;\;\;\;y=1\\ 1-\alpha \;\;\;\;\;otherwise \end{array}\right.$$

where $p_{t}$, is the model’s predicted probability for the true class. If the true class is y, $p_{t}$ is the predicted probability for class y. $\alpha_{t}$ is a weighting factor for the class, which helps balance the importance of positive versus negative examples. γ is a focusing parameter that adjusts the rate at which easy examples are down-weighted.

1. *If y* = 1 (positive class): *α_t_ = α*
2. *If y* = 0 (negative class): *α_t_ =* 1 – *α*
3. *α* is a scalar factor that adjusts the weight of positive examples. This helps to balance the contribution of positive and negative examples to the loss function.
4. Focusing parameter *γ*: It adjusts the rate at which easy examples are down-weighted. When *γ* = 0, the focal loss function becomes equivalent to the standard cross-entropy loss. As *γ* increases, the focus shifts more towards hard examples, making the model pay more attention to examples where the model’s prediction is incorrect or uncertain.

As mentioned in Equation (2), a combination of cross-entropy and the modulating factor ${\left(1-p_{t}\right)}^{\gamma }$ is used because it leads to better accuracy. In [36], a comparison between the cross-entropy w =0 and focal loss functions is presented. The term $\alpha_{t}{\left(1-p_{t}\right)}^{\gamma }$ is inversely proportional and depends on the value of $p_{t}$.

Focal loss functions are emerging as a method of cost-sensitive learning that balances the cross-entropy loss to train hard negative examples, including rare classes [36,37,38]. The authors confirmed the capability of focal loss functions in a detection process by considering imbalanced classes. Focal loss functions are suggested to change the shape and adjust cross-entropy to achieve better classification based on the loss function for dealing with imbalanced classes. Nevertheless, the utility of focal loss functions extends beyond the realm of detection tasks. Regardless of the number of classes and tasks, adjusting learning based on sample difficulty is effective [37].

Focal loss functions can significantly improve the performance of multi-class samples. This aligns with our theoretical hypothesis. The advantage of focal loss functions is dynamically adjusting the weight of mispredicted samples. This function can predict samples that occupy a small proportion of the entire dataset [39].

### 2.3. Related Works

Different intrusion detection models have tackled the challenge of imbalanced data [10,40,41,42,43,44,45,46,47,48,49,50,51]. These models often rely on data manipulation techniques, including oversampling and undersampling. In 2020, Zhang et al. [14] used a combination of SMOTE and Gaussian Mixture Clustering to balance the data and performed attack detection using convolutional neural networks (CNN). Their experimental results on the UNSW-NB15 and CICIDS2017 datasets demonstrated an accuracy of 96%. Yilmaz et al. [52] employed a Generative Adversarial Network (GAN) and a Multi-Layer Perceptron (MLP) for identifying rare attacks. Results on the UGR’16 dataset indicated that using a GAN for generating artificial data could moderately improve results compared to imbalanced original data. Panigrahi et al. [53] used Supervised Relative Random Sampling (SRRS) to reduce majority class data and employed the C4.5 decision tree algorithm for attack detection. Their results on the NSL-KDD and CICIDS2017 datasets reported accuracies of 99.96% and 99.95%, respectively. In 2022, Ding et al. [2] employed a combination of Generative Adversarial Networks (GANs) and nearest neighbor techniques to handle imbalanced data. Their results across the KDD99, UNSW-NB15, and CICIDS2017 datasets achieved accuracies ranging from 90% to 95%. Huang et al. [54] based their model on a GAN and used convolutional neural networks for classifying attacks. Results on the NSL-KDD, UNSW-NB15, and CICIDS2017 datasets demonstrated accuracies ranging from 82% to 99%. FU et al. [5] used ADASYN, an improved version of SMOTE, to balance the data and performed attack detection using Bidirectional Long Short-Term Memory (BiLSTM). They achieved an accuracy of 90.73% on the NSL-KDD dataset. Yan et al. [55] employed density-based clustering with DPeak for data balancing. Results on the KDD99-2D, CIC-IDS2017, and ISCXID2012 datasets reported accuracies between 79% and 94%. Reem Alshamy et al. [21] used a combination of Random Forest and SMOTE. Their results on the NSL-KDD dataset showed the success of their model with an accuracy of 99.89%. Malik AL-Essa [56] used XGBoost and SMOTE. Similarly, Huang Le et al. [8] also used the XGBoost algorithm. Results on the X-IIoTDS and TON_IoT attack datasets showed an F1 score of around 99%.

In 2022, Cao et al. [57] applied convolutional neural networks and a Gated Recurrent Unit (GRU) to perform attack detection. Results across the UNSW_NB15, NSL-KDD, and CIC-IDS2017 datasets yielded accuracies of 86.25%, 99.69%, and 99.65%. Balyan et al. [58] employed genetic algorithms, Particle Swarm Optimization with Estimation of Distribution (EGA-PSO), and Improved Random Forest (IRF). EGA-PSO was used to increase samples of minority class data. Their evaluation of the NSL-KDD dataset showed an accuracy of 98%. Jung et al. [59] introduced an approach to tackle imbalanced data challenges using SMOTE and a changed neural network that increases the minority class and removes noisy data. Their method showed promising results in evaluations of the PKDD2007 and CSIC2012 datasets. Dar et al. [60] used a Variational Autoencoder and a Multi-Layer Perceptron (MLP) to address imbalanced data. Their evaluation of HDFS data demonstrated an F1 score of 97%. Kayo et al. [61] employed ADASYN for balancing and conducted attack classification using convolutional and Bidirectional GRU (BiGRU) neural networks. They compared their new method with their previous approach [57] and showed that the new method outperformed the previous one. In the UNSW_NB15, NSL-KDD, and CIC-IDS2017 datasets, the new method achieved accuracies of 85.55%, 99.81%, and 99.70%, respectively. In 2022, Tareq et al. [62] used two intelligent network models, namely DenseNet and Inception Time, for cyber-attack detection. They evaluated their models on the ToN-IoT, Edge-IIoT, and UNSW2015 datasets. Their best result was a 99.9% accuracy for Windows 10 with DenseNet. In 2023, Thockchom et al. [63] proposed a group-based learning approach for intrusion detection that outperforms individual classifiers. Their model utilizes lightweight machine learning models and Chi-square feature selection. This method proves effective in imbalanced datasets, reducing the cost of misclassifications, and demonstrates its utility in intrusion detection systems. In another study in 2023, Sarvar et al. [64] presented a machine learning-based anomaly detection approach for smart homes using various classifiers. Their experiments and evaluations on the BoT-IoT dataset resulted in accuracy, recall, and F1 scores of 0.98, 0.96, and 0.96, respectively.

The aforementioned studies reveal that most imbalanced data-focused methods rely on data manipulation, often attempting to augment the number of minority class data through artificial data generation. Data manipulation presents its own set of challenges. In most network traffic datasets, the imbalance is severe, and generating data for minority classes results in increased data volume. This, in turn, leads to issues such as increased computational complexity and model inefficiency in real-world scenarios. On the other hand, generating a large volume of data for the minority class may not ensure data quality, as the outcomes of many algorithms in the SMOTE family depend on the proper determination of an appropriate neighbor count ‘k.’. If the number of neighbors is not correctly determined for SMOTE family algorithms, it can result in the generation of low-quality synthetic data, consequently decreasing the performance of intrusion detection models. Therefore, data manipulation is not an ideal solution for addressing the challenge of imbalance. In this context, considering the above discussions, this article introduces an innovative approach to control and manage data imbalances. The primary objective of this approach is the detection of rare and minority attacks without data manipulation.

## 3. Proposed Architecture for Deep Binary Intrusion Detection

**Figure 2:**
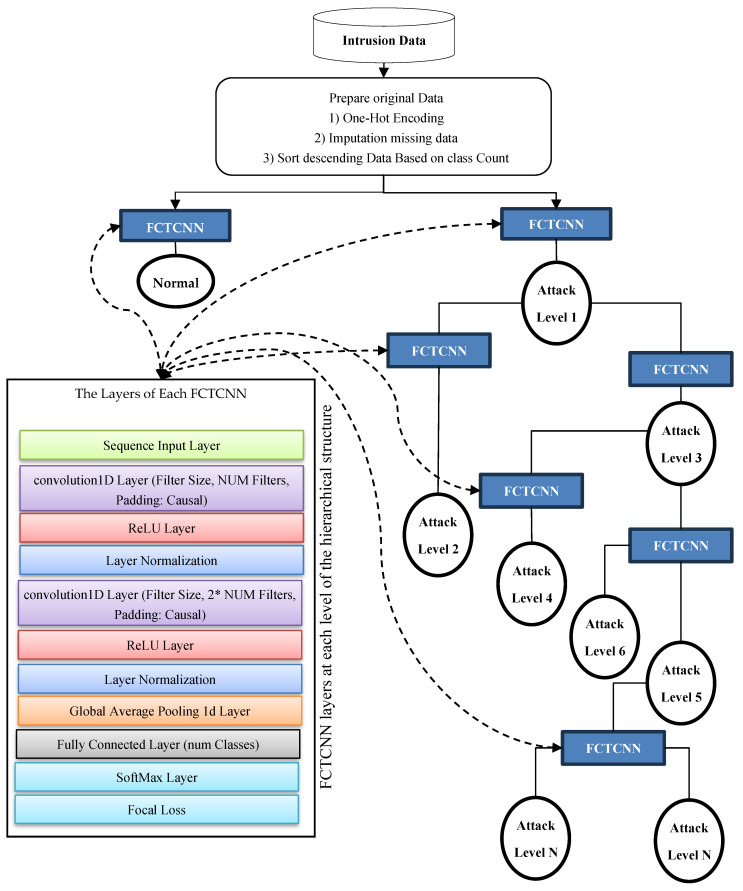
Conceptual diagram of the proposed FCTCNN model.

In this paper, a deep binary architecture named focal causal temporal convolutional neural network (FCTCNN) is proposed for detecting rare attacks in IoT and Industrial IoT environments, aimed at addressing the challenge of imbalanced data. As illustrated in Figure 2, this model defines a deep binary architecture comprising causal temporal convolutional networks. At each level, attacks with similar frequencies are identified and mitigated. One of the advantages of the proposed structure is its reduction in computational and temporal complexity due to the deepening of the architecture. Specifically, rare attacks with minimal sample sizes are detected at the leaf level, while majority attacks are detected at the root level. Consequently, by deepening the architecture, the model’s complexity is reduced and the learning time is significantly decreased. The subsequent sections provide a comprehensive explanation of the proposed model’s stages.

In Figure 2, the proposed deep causal hierarchical architecture is illustrated. This structure is composed of causal convolutional networks (depicted as blue squares in Figure 2), with the constituent layers of these networks shown on the left side of the figure. Briefly, in this method, network traffic data are sorted in descending order based on the number of samples in each class. At the highest level of the architecture, the first type of attack, which has the largest number of samples, along with the Normal class, which typically has the highest number of samples in network traffic data, are processed using the proposed method. These two instances are considered the majority classes in the data, which often leads to inadequate learning with conventional deep learning methods. However, the hierarchical model proposed in this study addresses this issue by defining a deep binary structure at each level.

The proposed network performs the learning process with these two labeled datasets and generates its predictions. At the subsequent level, data from these two classes are removed from the entire dataset, and two additional types of attacks with equal sample sizes are introduced to the model for further learning and classification. At each level, classified data are removed, and the remaining data from the database are presented to the network in pairs according to the sample sizes of each class until all data are classified.

As the volume of data decreases at each level of the model, and majority classes are eliminated at the first level of the tree structure, the number of data points decreases as the depth of the tree structure increases. Consequently, the model trains and predicts more efficiently with smaller datasets. In this model, the levels of the proposed structure vary based on the number of attacks for each database and are automatically adjusted to form a suitable structure for each dataset. The subsequent sections provide a more detailed explanation of the model’s stages.

### 3.1. Data Preprocessing

**Figure 3:**
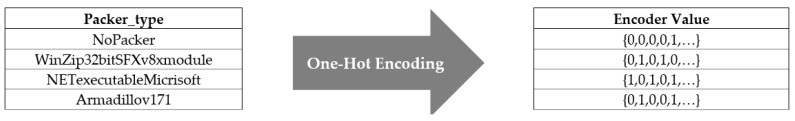
Transforming nominal data into numeric data using One-Hot encoding [65].

In network traffic data, there are many nominal features, such as port names, that machine learning methods cannot directly process. Therefore, a solution is required to convert these data into numerical values without sacrificing model accuracy. In our model, the One-Hot encoding technique is used to transform all nominal data into numerical data. Certainly, as illustrated in Figure 3, distinct variables within each nominal attribute are identified, and for each unique variable, a binary feature is incorporated into the dataset.

### 3.2. Handling Missing Values

The presence of missing values in network traffic data is another challenge that, if left unaddressed, can reduce the effectiveness of the intrusion detection model. Failing to adopt an appropriate strategy for managing missing data compromises the model’s performance. In this research model, the nearest neighbor technique is used to manage missing data, and the missing values are replaced with the values corresponding to the nearest neighbors based on Euclidean distance. Specifically, for each missing data point, the first Euclidean nearest neighbor is found, and the missing value is replaced with the corresponding value from the complete data.

### 3.3. Causal Temporal Binary Convolutional Architecture

The causal convolutional network defined in this study, as illustrated in Table 1, is composed of 11 layers and has over 42,000 learnable parameters.

The layers of this network are described as follows:

- Input Layer: Receives data in a sequential format with dimensions of 1.
- Second Layer: A 1-dimensional convolutional layer, where 64 filters of size [1 × 5] are defined, with a stride of 1. This means that each filter has a width of 5 units and a height of 1 unit. In practical terms, the filter slides over the input data, which is structured as a 1-dimensional sequence, to detect features of length 5. The stride is set to 1. This parameter controls how much the filter moves (or “strides”) across the input data. With a stride of 1, the filter shifts by one unit at a time. This results in a convolution operation that processes every possible position of the filter over the input sequence.
- Third Layer: A ReLU activation function layer, which maps negative values to zero using the relation *ReLU(x)* = max (0, *x*). This layer passes positive values unchanged to the next layer.
- Fourth Layer: A normalization layer that normalizes the input data across channels and applies a learnable affine transformation with parameters. Normalizing across channels typically involves adjusting the mean and variance of the data in each channel to ensure that they are standardized. This helps stabilize and accelerate the training process by reducing internal covariate shift. After normalization, the layer applies a learnable affine transformation. This involves two operations: Scaling: Each normalized value is multiplied by a learnable parameter called the scale factor (*γ*). Shifting: Each normalized value is then shifted by another learnable parameter called the shift factor (*β*).

This affine transformation allows the network to adjust and fine-tune the normalized data, making the normalization process more flexible and suitable for learning complex patterns. The parameters *γ* and *β* are learned during training and help the network recover any potential loss of representational power caused by the normalization.

- Fifth Layer: A second convolutional layer with 128 filters, where the stride and filter dimensions are consistent with those of the first convolutional layer in the defined architecture.
- The sixth and seventh layers: These are a ReLU activation layer and a normalization layer, respectively, as previously described.
- Eighth Layer: This is a global average pooling layer that calculates the average along the 1D input length dimension. It computes the average value across the entire length of the 1D input dimension. Essentially, it takes the mean of all the values in each feature map along the sequence dimension. If the input to this layer is a 1D sequence of length *L*, the output for each feature map will be a single value representing the average of all *L* values in that feature map. The GAP layer significantly reduces the dimensionality of the data. By summarizing the entire sequence into a single value per feature map, it transforms a potentially large number of activations into a compact vector. This reduction helps in minimizing computational costs and model complexity. By summarizing the feature maps into a single value per feature map, the GAP layer helps in reducing the risk of overfitting. It does this by eliminating the fine-grained details that might cause the model to memorize the training data rather than generalize well.
- Ninth Layer: This is a fully connected layer that multiplies the synaptic weights with the input data and computes the sum.
- Final Layers: The architecture also includes a SoftMax layer and a classification layer that utilizes the focal loss function, which is effective in addressing data imbalances. The focal loss function is used in this layer to address class imbalances by focusing more on hard-to-classify examples and reducing the impact of well-classified examples. The SoftMax layer is used to produce probability distributions for the network’s output. It converts the raw scores (logits) from the previous layer into probabilities by exponentiating the scores and then normalizing them. Specifically, for each class, the SoftMax function computes the probability *P*(*y_i_*) as follows:

(3) $$P\left(y_{i}\right)=\frac{e^{Z_{i}}}{\sum_{j}e^{Z_{j}}}$$

where $Z_{i}$ represents the score for class i and the denominator is the sum of exponentiated scores for all classes. This ensures that the output probabilities are positive and sum up to 1, making them suitable for classification tasks.

After establishing the causal convolution layers, which act as deep nodes at each level of the proposed hierarchical structure, the learning phase of the hierarchical structure must be executed. To train the network, the data are first divided into training and testing subsets at an 80% and 20% ratio, respectively. Subsequently, the network traffic data are sorted in descending order based on frequency (number of samples per class) as illustrated in Table 2. For the first level of the tree structure, data from each dataset are presented to the network with two labels: Normal and Attack. The model is trained once with the aforementioned data, and all data associated with the Normal class are removed from the dataset.

Following the initial training, the code progresses with a hierarchical approach to address different attack classes. The model is iteratively refined by focusing on different attack categories and removing benign (Normal) samples and classes with fewer samples. The model is retrained and evaluated for each level, with training settings adjusted based on the number of classes and remaining data. Finally, the network’s performance is assessed using ROC metrics and confusion matrices for each attack category, providing a comprehensive view of the model’s effectiveness across various attack levels. This hierarchical approach ensures that the model is designed to manage imbalanced datasets effectively, focusing on detecting less frequent but critical types of attacks.

**Figure 4:**
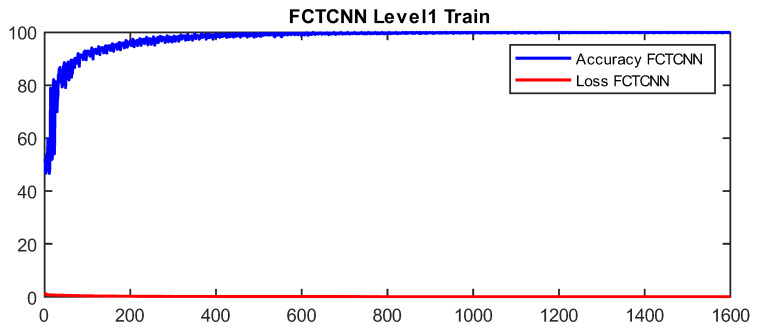
Learning curve at Level 1 in the proposed architecture in the TON_IIOT dataset.

**Figure 5:**
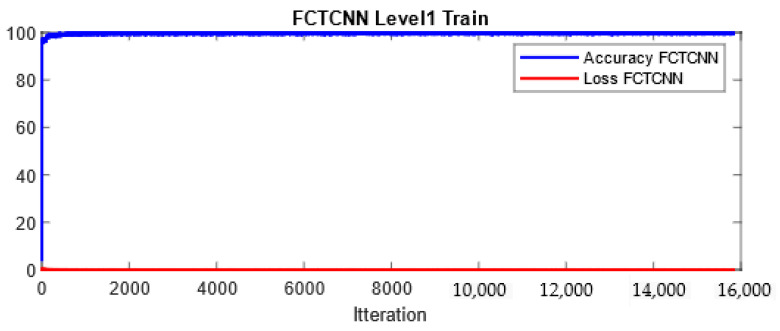
Learning curve at Level 1 in the proposed architecture in the UNSWNB-2015 dataset.

In more detail, data are classified into two classes at the first level of our architecture: Normal and Non-Normal. Before completing learning and attack prediction in the testing phase, data corresponding to the Normal class are removed from the training dataset. At the second level of the proposed architecture, the first attack, which is larger compared to other attacks, is considered the primary class, and other attacks are treated as secondary attacks for classification. This process continues until the rarest attacks in each dataset are reached. At the final level of the proposed architecture, the two rarest attacks in the dataset are classified. As mentioned, at each level of the deep binary architecture, the detected attack is removed from the list of attacks, which in turn significantly reduces the computational complexity of the model. In our model, the Adam optimization function is used for training the network, and the number of epochs is increased from 50 to 200 with the increasing depth of the network. Additionally, the mini-batch size is reduced from 1024 at the root node to 16 at the leaf level of the proposed architecture. Figure 4 and Figure 5 show a sample learning curve of the proposed network at Level 1 (root node) for classifying data into Normal and Non-Normal (Attack) classes in the TON_IIOT and UNSWNB2015 datasets.

## 4. Evaluation and Results Analysis

For evaluating the model in this article, precision, recall, accuracy, specificity, average f, Matthews’ correlation coefficient, Cohen’s kappa, time, and ROC curve metrics were used. These metrics were calculated from Equations (4) to (11).

(4) $$Accuracy=\frac{TP+TN}{TP+TN+FP+FN}$$

(5) $$Recall=\frac{TP}{TP+FN}$$

(6) $$F_{measure}=\frac{2\times Precision\times Recall}{Precision+Recall}$$

(7) $$Time\left(s\right)=end_{t}-Start_{t}$$

(8) $$Specificity=\frac{TN}{TN+FP}$$

(9) $$Presicion=\frac{TP}{TP+FP}$$

(10) $$MCC=\frac{TP\;*\;TN-FP\;*\;FN}{\sqrt{\left(TP+FP\right)\;\left(TP+FN\right)\;\left(TN+FP\right)\;\left(TN+FN\right)}}$$

(11) $$K=\frac{2*(TP\;*\;TN-FP\;*\;FN)}{\left(TP+FP\right)*\;\left(TP+FN\right)*\;\left(TN+FP\right)*\;\left(TN+FN\right)}$$

In the equations above, the variable TP refers to data points that are actually positive and have been correctly predicted as positive by the model. Similarly, the variable TN refers to data points that are actually negative and have been correctly predicted as negative by the model. On the other hand, the variable FP refers to data points that are actually negative but have been incorrectly predicted as positive by the model, while FN refers to data points that are actually positive but have been incorrectly predicted as negative by the model.

**Figure 6:**
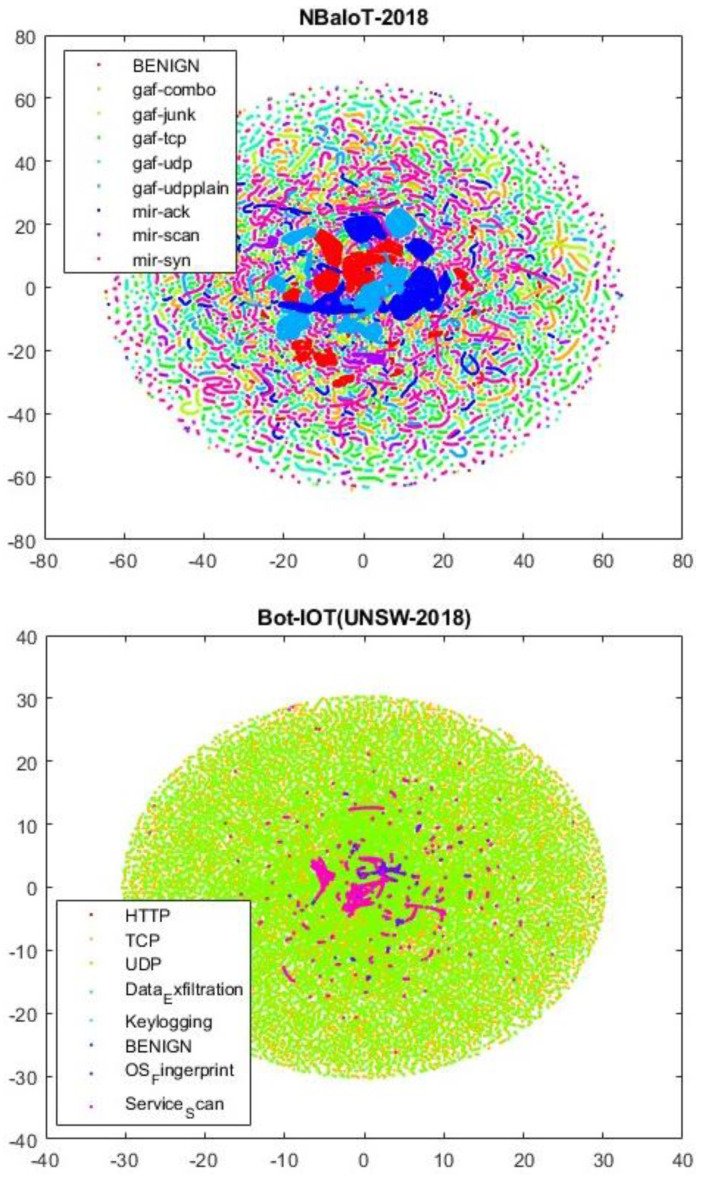
A two-dimensional t-NSE representation of the BoT-IoT and NBaIoT datasets.

The evaluation of models on diverse datasets is available, each with various types of attacks. Considering that the behavior of models can differ from one dataset to another, a comprehensive examination of the model’s performance was conducted using five different datasets, as follows:

— TON-IIOT dataset: This dataset includes heterogeneous data sources collected from telemetry datasets of IoT and IIoT sensors, operating system datasets for Windows 7 and 10, etc. [66].
— UNSW-NB15 dataset: This dataset consists of four sections, and Section 1 is used in this article. It comprises 47 features, two columns for attack labels, and over 700,000 records. The dataset includes nine types of attacks and a normal class [67].
— BoT-IoT dataset: Designed in the Cyber Range laboratory at UNSW Canberra, this dataset simulates a real network environment. It incorporates a blend of normal and botnet traffic, presenting source files in various formats, including original pcap files, generated argus files, and CSV files. This dataset comprises over 72,000,000 records, encompassing attacks like DDoS, DoS, OS and Service Scan, Keylogging, and Exfiltration Data [68].
— CICIDS-2017 dataset: This dataset includes six attack types and a normal class, composed of 80 features and two columns for attack labels, totaling 745,423 records. It covers attacks such as SQL injection, brute force, DDoS, SSH-Patator, Port-Scan, and more [69].
— NBaIoT dataset: Focused on botnet attacks in the Internet of Things, this dataset is gathered from nine commercial IoT devices. It includes five BASHLITE and five Mirai attacks, featuring 115 features and 701,000 records [70]. In Figure 6, a two-dimensional view of the BoT-IoT and NBaIoT datasets is presented.

For model implementation, MATLAB 2023a was used on a system with nine processors, 32GB RAM, and an Nvidia RTX 3080 GPU. Data rates of 20% for testing and 80% for training were established. Network learning parameters included the Adam optimization function, initial learning rate of 0.01, Max Epoch count ranging from 50 to 200, and minibatch sizes ranging from 1024 to 16.

### 4.1. Results Analysis in the UNSW-NB2015 Dataset

Considering the imbalance challenge in this study, results for each dataset are reported on classes to assess the model’s performance on rare classes. Table 3 presents the detection results of our model for attack types in comparison with two models [2,71], and the LSTM network in [72] on the UNSW-NB2015 dataset.

UNSW-NB2015 is one of the datasets with a severe data imbalance issue. In this dataset, as indicated in Table 2, four types of attacks, such as Backdoors, have around 500 or fewer data samples, significantly lower than larger attacks. Balancing this data issue through data manipulation and synthetic data generation is unlikely to be very successful and may lead to computational complexity. However, our model, as shown in Table 3, effectively handles the detection of rare attacks without data manipulation.

In the case of the four minority attacks in this dataset, namely ‘Backdoors’, ‘Analysis’, ‘Shellcode’, and ‘Worms’, our deep binary structure has achieved very good results compared to other methods. In the three attacks ‘Analysis’, ‘Shellcode’, and ‘Worms’, our model has achieved 100% results across all metrics, whereas other models have obtained 0% results in these three attack types. In the ‘Analysis’ attack, the LSTM recurrent network [72] has failed in all metrics, indicating that even very deep networks face severe challenges in detecting extremely rare attacks if the data imbalance issue is not addressed. In this attack, a combination of k-nearest neighbors and an adversarial generative network achieved an F1-score of 83.17%, demonstrating that generating synthetic data using deep networks for minority classes can somewhat control the data imbalance challenge. However, this method still showed a weaker recall and average F1-score compared to our proposed deep model. In the ‘Shellcode’ attack, one of the rarest attacks in this dataset with around 223 data samples, other models like the LSTM deep network failed to yield results and completely failed. However, the KNN-GAN, which focuses on generating artificial data with a neural network, ranked second after the proposed model. The ResNet50-Oversample model, despite using oversampling and generating artificial data, still presented results below 20%, indicating that artificial data generation may not be very successful in this context.

**Figure 7:**
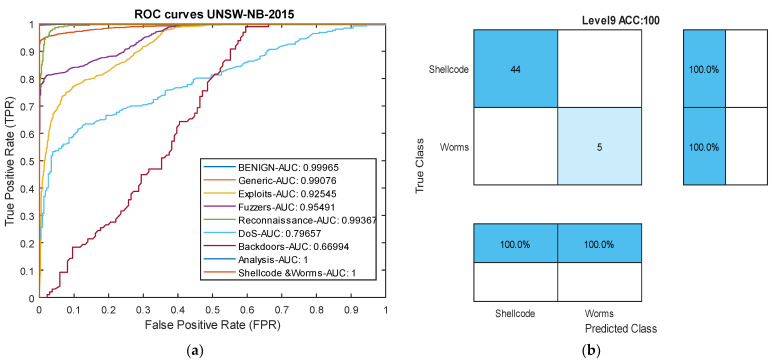
ROC plot and confusion matrix from the UNSW-NB2015 dataset. (**a**) ROC curve. (**b**) Level 9 confusion matrix: Worms and Shellcode attacks.

Among the attacks in this dataset, our model struggled to succeed only in the ‘Exploits’ and ‘Generic’ attacks compared to other models. Nevertheless, in these two attack types, the results were still very close to other models. Overall, the results in Table 3 indicate that the proposed deep binary architecture based on causal temporal convolutional networks has performed well in detecting attacks across most classes, achieving an over 10% increase in accuracy compared to other methods. Figure 7 presents the ROC curve and confusion matrix of the proposed model in the UNSW-NB2015 dataset.

### 4.2. Evaluation of Results in the CIC-IDS2017 Dataset

**Figure 8:**
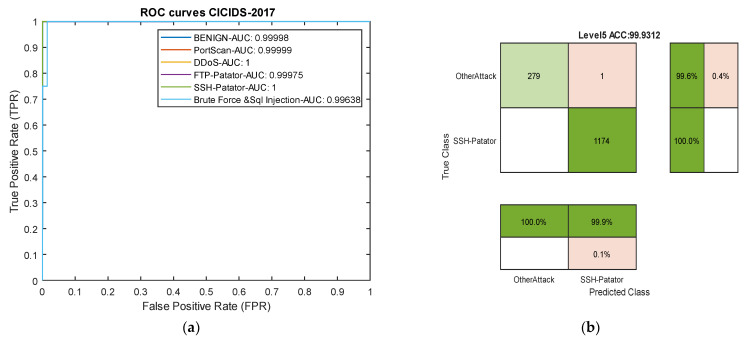
Receiver Operating Characteristic (ROC) curve and confusion matrix for the CICIDS-2017 dataset. (**a**) ROC curve. (**b**) Level 5 confusion matrix for the SSH-Patator attack.

Table 4 compares the results of our model with [63] on the CIC-IDS2017 dataset. In this dataset, the group model proposed in [63] completely failed in detecting two types of attacks. Meanwhile, our proposed model, due to its transformation of multi-class classification into binary classification, achieved an overall precision of over 99% for all attacks. Considering the frequency of attacks in their classification has allowed the model to effectively address the issue of data imbalance. In this dataset, the model achieved an average precision of 99.84% for all attacks, while the group method [63] attained a precision of 67.52% for all attacks. These results indicate that the introduction of the new architecture without data manipulation has led to success in detecting both rare and common attacks, surpassing 30%. Figure 8 presents ROC curve and one confusion matrix of the proposed model in this dataset.

### 4.3. Results Analysis in the TON-IIOT Dataset

**Figure 9:**
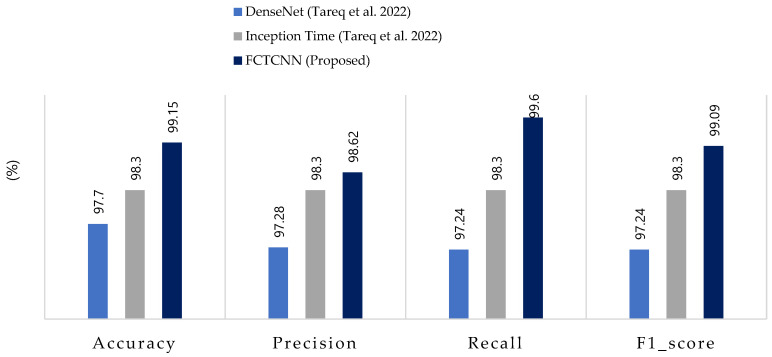
Comparison of accuracy, precision, recall, and F1-score for the proposed method, compared to methods by DenseNet, Inception Time and FCTCNN on the Industrial TON-IIOT dataset [62].

**Figure 10:**
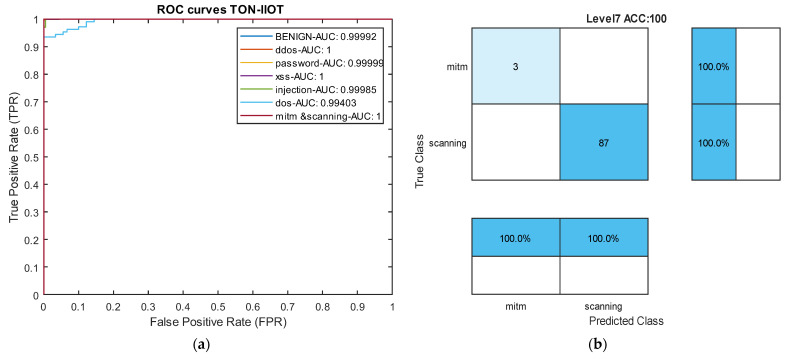
ROC Curve and confusion matrix in the TON-IIOT dataset. (**a**) ROC curve. (**b**) Level 7 confusion matrix: mitm and scanning attacks.

Table 5 reports the results of our model in the heterogeneous Industrial Internet of Things (IIoT) dataset TON-IIOT and the average results across all classes are compared with the model by Tareq et al. [62] in Figure 9. In this dataset, four types of attacks—Injection, DOS, MITM, and Scanning—are rare, and the model achieved an average accuracy of over 97% and recall of over 99% for all four classes. Particularly, for the very rare MITM and Scanning attacks, the model demonstrated 100% accuracy, recall, and F1-scores. These results indicate that the proposed model can effectively control rare attacks across various datasets. The ROC curve diagram and confusion matrix for two rare attacks for the proposed model in this dataset are presented in Figure 10.

Comparing the results with two deep neural networks presented by Tareq et al. [62] shows a 2.3% increase in overall accuracy. Furthermore, in precision, recall, and average F1-score, our model outperformed those of Tareq et al. by 1.66%, 3.66%, and 2.64%, respectively. These comparative results highlight that our model has achieved greater success in this dataset compared to the latest existing methods. The nature of the temporal convolutional network contributes to the model’s success in detecting various types of attacks. The impact of the focal loss function cannot be ignored in this success.

### 4.4. Results Analysis in the BoT-IoT (UNSW_2018) Dataset

**Figure 11:**
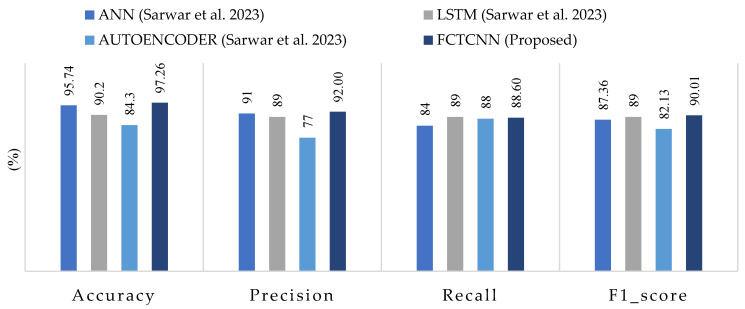
Comparison of accuracy, precision, recall, and F1-score for the proposed method, compared to methods by ANN, LSTM, AUTOENCODER and FCTCNN on the Industrial BOT-IOT dataset [64].

Table 6 presents the results of our model for each type of attack in the BoT-IoT dataset. Figure 11 compares the average results across all attacks using the approach proposed by Sarwar et al. [64]. The results of this evaluation show that the model successfully detected Keylog attacks with 94% accuracy and Data Exfiltration attacks with 50% accuracy, even though these attacks are rare, consisting of 73 and 6 data samples, respectively. Most studies struggle to predict these two types of attacks due to their rarity.

**Figure 12:**
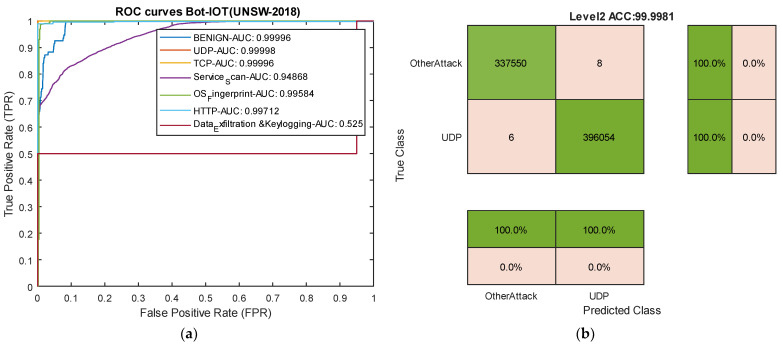
ROC curves for two attack types and confusion matrix for UDP attacks in the BoT-IoT dataset. (**a**) ROC curves. (**b**) Level 2 confusion matrix for UDP attacks.

Comparing the results with the methods proposed by Sarwar et al. reveals that, among the deep learning methods used by Sarwar, the artificial neural network (ANN) model outperformed the recurrent neural network (RNN) and Autoencoder. In comparison with the ANN model in this dataset, our model improved accuracy by 1.52%, precision by 1%, and recall and F1-score by 4.60% and 2.65%, respectively. The successful classification of attacks with similar frequency and usage of an appropriate loss function for training the proposed deep neural network, along with the incorporation of temporal convolutional layers, contributes to the model’s success compared to existing methods on various datasets. The ROC curve diagram and confusion matrix for two rare attacks for the proposed model in this dataset are presented in Figure 12.

### 4.5. Results Analysis in the NBaIoT-2018 Dataset

**Figure 13:**
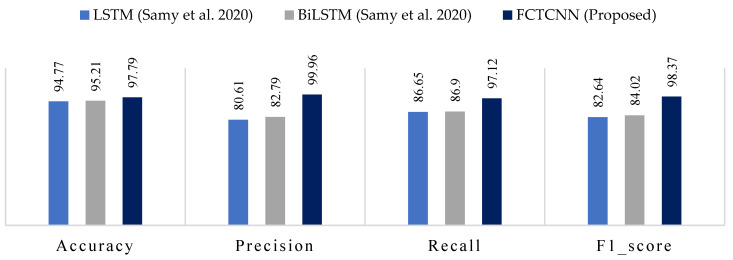
Comparison of accuracy, precision, recall, and F1-score for the proposed method, compared to methods by LSTM, BiLSTM and FCTCNN on the Industrial NBaIoT-2018 dataset [72].

In the final evaluation category, Table 7 reports the results of our model for detecting different attack types in the NBaIoT-2018 dataset. Figure 13 compares the results with models proposed by Samy et al. [72]. The results of this assessment demonstrate that a well-defined architecture leads to the complete success of our model in handling both common and rare attacks. For instance, the LSTM and BiLSTM methods presented by Samy et al. utterly failed in detecting the Gaf-Tcp attack, yielding 0 in all metrics. In contrast, our cutting-edge deep architecture achieved over 99% accuracy in this attack. While this dataset is not as imbalanced as others in this study, the proposed temporal convolutional binary architecture still provides very promising results. Among the three models, LSTM, BiLSTM, and FCTCNN, LSTM yielded the weakest results. Compared to LSTM, our model increased accuracy by over 19% and recall by over 10%.

In the second rank after our model, BiLSTM exhibited improved accuracy by over 17% and recall by over 10%. In the F1-score metric, our deep neural network outperformed the presented recurrent neural networks in [72] by 14 to 15%. The results across all five datasets indicate the success of our model compared to existing methods.

**Figure 14:**
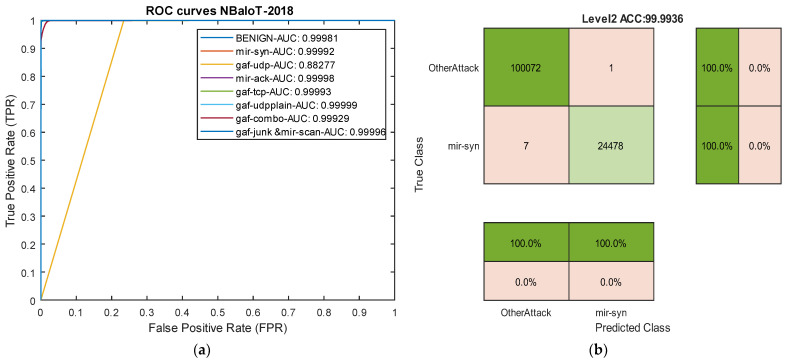
ROC curves and confusion matrix for two attack types in the NBaIoT dataset. (**a**) ROC curves. (**b**) Level 2 confusion matrix for the Mir-Syn attack.

The results from various experiments highlight that a properly designed deep architecture can outperform data-centric methods that address the imbalance issue using artificial data generation. Overall, the findings in this paper, across five different datasets with various attacks, demonstrate that the proposed cutting-edge binary architecture based on causal temporal convolutional networks can effectively control the challenge of detecting rare attacks. Two sample ROC curve diagrams for our model in the NBaIoT dataset are presented in Figure 14.

### 4.6. Analysis of Time Complexity across All Data

**Figure 15:**
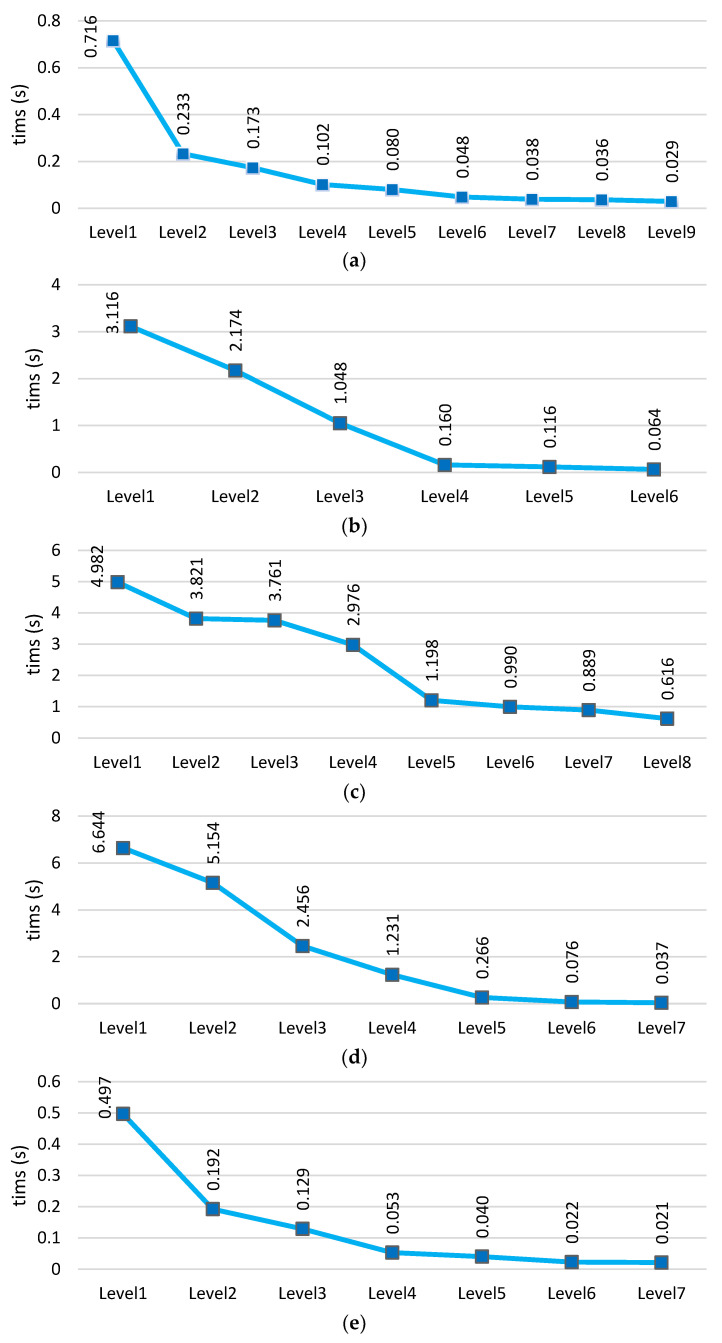
Prediction time comparison of the proposed deep hierarchical model across five datasets: (**a**) UNSW-NB15, (**b**) CICIDS2017, (**c**) N_BaIoT2018, (**d**) BoT-IoT, and (**e**) TON-IIOT.

As stated in the section introducing the proposed method, in our approach, increasing the level of the hierarchical structure leads to a reduction in computational and time complexity. This is because data from previous levels are eliminated at each level in this method, resulting in minimal data available for learning and prediction in the final levels. Consequently, the model can reduce time at the final levels. Figure 15 shows the time charts across five different datasets for each level of the model. These charts clearly demonstrate that the model’s time decreases from the beginning towards the end (the final levels). This reduction occurs because data are eliminated at each level.

The analysis shows that despite the hierarchical structure of the model, the time has not increased due to the reduction in data at each level. Compared to other deep neural networks, our method achieves lower prediction times. Recurrent neural networks like LSTM and BiLSTM have greater time complexity compared to our method, primarily due to the internal structure of their hidden cells, which involves loops and feedback. On the other hand, the KNN algorithm, known as a lazy learning algorithm, does not perform computations during the training phase but only during the evaluation phase. Consequently, its prediction time is longer compared to our model. This evaluation clearly demonstrates that the proposed structure has a favorable complexity compared to other deep neural networks. Table 8 presents the comparative results of average time (seconds) across five datasets.

## 5. Conclusions

In intrusion detection systems, class imbalances across different classes are a major challenge. The issue of class imbalance in intrusion detection datasets often confounds engineers and researchers. Despite the high accuracy achieved by shallow machine learning and deep neural network models, NIDS models suffer from high false positive rates and lower intrusion detection rates due to imbalanced datasets. Data imbalance in datasets refers to conditions where class distributions are disproportionately represented. This situation arises when a majority class significantly outweighs a minority class. Most existing studies have addressed this challenge using oversampling and undersampling techniques. Although these methods have been somewhat successful, they also introduce problems such as increased data volume and computational complexity. Therefore, this paper presents an innovative deep binary model based on deep convolutional neural networks with focal loss to address the issue of imbalanced data. In this model, multi-class classification is converted into a sequential binary classification problem. Evaluations on five different network traffic flow datasets show that the proposed model effectively addresses the problem of rare attacks and demonstrates the capability to detect them. Our model has not failed in any of the five studied datasets or attacks and has consistently achieved over 99% accuracy in identifying rare attacks. Future work could include the use of wrapper-based feature selection methods based on metaheuristic algorithms to further enhance the results. Additionally, a competitive framework could be introduced where two types of deep architectures compete at each level, with the winning model receiving more data in subsequent levels.

## Figures and Tables

**Table 1:** The constituent layers of the proposed architecture.

	Name	Type	Activations	Learnable Properties
1	SEQUENCE INPUT Sequence input with 1 dimension	SEQUENCE INPUT	1(C)×1(B)×1(T)	-
2	CONV1-D_1 64 × 5 convolutions with stride 1 padding “causal”	1-D CONVOLUTION	64(C)×1(B)×1(T)	Weights 5×1×64 Bias 1×64
3	ReLU_1	ReLU	64(C)×1(B)×1(T)	-
4	LAYERNORM_1	LAYER NORMALIZATION	64(C)×1(B)×1(T)	Offset 64×1 Scale 64×1
5	CONV1-D_2 128 × 5 convolutions with stride 1 padding “causal”	1-D CONVOLUTION	128(C)×1(B)×1(T)	Weights 5×64×128 Bias 1×28
6	ReLU_2	ReLU	128(C)×1(B)×1(T)	-
7	LAYERNORM_2	LAYER NORMALIZATION	128(C)×1(B)×1(T)	Offset 128×1 Scale 128×1
8	GLOBALAVGPOOL1D 1-D Global average Pooling	1-D GLOBAL AVERAGE POOLING	128(C)×1(B)	-
9	FC 2 fully connected layer	FULLY CONNECTED	2(C)×1(B)	Weights 2×128 Bias 2×1
10	SOFTMAX	SOFTMAX	2(C)×1(B)	-
11	FOCALLOSS	FOCAL LOSS LAYER	2(C)×1(B)	-

**Table 2:** Sorting of attacks in descending order in 5 datasets.

NBaIoT-2018		ToN_IIoT		BoT-IoT (UNSW-2018)		CICIDS-2017		UNSW-NB2015	
Count	Attack	Count	Attack	Count	Attack	Count	Attack	Count	Attack
122,573 105,874 102,195 92,141 81,982 59,718 29,849 29,068	‘mir-syn’ ‘gaf-udp’ ‘mir-ack’ ‘gaf-tcp’ ‘gaf-udpplain’ ‘gaf-combo’ ‘mir-scan’ ‘gaf-junk’	4608 3628 1269 612 525 447 15	‘DDos’ ‘Password’ ‘XSS’ ‘injection’ ‘Dos’ ‘Scanning’ ‘Mitm’	1,981,230 1,593,180 73,168 17,914 2474 73 6	‘UDP’ ‘TCP’ ‘Service_Scan’ ‘OS_Fingerprint’ ‘HTTP’ ‘Keylogging’ ‘Data_Exfiltration’	158,930 128,027 7938 5897 1507 21	‘PortScan’ ‘DDoS’ ‘FTP-Patator’ ‘SSH-Patator’ ‘Brute Force’ ‘Sql Injection’	7522 5409 5051 1759 1167 534 526 223 24	‘Generic’ ‘Exploits’ ‘Fuzzers’ ‘Reconnaissance’ ‘DoS’ ‘Backdoors’ ‘Analysis’ ‘Shellcode’ ‘Worms’

**Table 3:** Performance comparison of deep models on the UNSW-NB2015 dataset.

Attack Name	Model	Precision	Recall	F1-Score	MCC	Kappa	Specificity	AUC
Normal	KNN-GAN (2022) [2]	-	94.39	94.03	-	-	-	-
	ResNet50 (2021) [71]	88.7	75.04	81.30	83.10	83.73	90.21	97.21
	ResNet50-Oversample (2021) [71]	92.3	64.6	76	72.54	71.92	93.10	96.44
	LSTM (2020) [72]	99.6	99.5	99.54	93.3	93.2	94.21	0.9
	FCTCNN	99.85	99.76	99.81	93.94	93.93	95.33	0.99
Backdoor	KNN-GAN (2022) [2]	-	49.17	53.23	-	-	-	-
	ResNet50 (2021) [71]	2.7	0.2	0.37	0	0	0	0
	ResNet50-Oversample (2021) [71]	6.3	39.3	10.85	0	0	0	0
	LSTM (2020) [72]	0	0	0	0	0	0	0
	FCTCNN	43.28	27.10	33.33	17	15.9	73.04	0.66
Analysis	KNN-GAN (2022) [2]	-	71.40	83.17	-	-	-	-
	ResNet50 (2021) [71]	0	0	0	0	0	0	0
	ResNet50-Oversample (2021) [71]	2.3	8	3.57	0	0	0	0
	LSTM (2020) [72]	0	0	0	0	0	0	0
	FCTCNN	100	100	100	100	100	100	1
Fuzzers	KNN-GAN (2022) [2]	-	71.73	77.32	-	-	-	-
	ResNet50 (2021) [71]	24.1	30.4	26.88	7.1	7.9	33.33	0.491
	ResNet50-Oversample (2021) [71]	22.8	41.5	29.43	7.3	7.9	34.21	0.499
	LSTM (2020) [72]	39.6	63.3	48.72	10.1	11.21	69.21	0.51
	FCTCNN	96.95	79.27	87.22	76.32	74.81	96.98	95.49
Shellcode	KNN-GAN (2022) [2]	-	81.64	41.55	-	-	-	-
	ResNet50 (2021) [71]	32.7	8.7	13.74	0	0	0	0
	ResNet50-Oversample (2021) [71]	6.2	76.2	11.46	0	0	0	0
	LSTM (2020) [72]	0	0	0	0	0	0	0
	FCTCNN	100	100	100	100	100	100	1
Reconnaissance (Probe)	KNN-GAN (2022) [2]	-	89.73	85.53	-	-	-	-
	ResNet50 (2021) [71]	62.9	70.5	66.48	44.63	44.61	87.80	0.736
	ResNet50-Oversample (2021) [71]	65.6	60.7	63.05	45.54	45.1	88.21	0.745
	LSTM (2020) [72]	64.9	41.6	50.70	44.63	43.42	87.53	0.715
	FCTCNN	98.48	97.42	97.95	95.10	95.09	97.87	0.9936
Exploits	KNN-GAN (2022) [2]	-	76.92	74.41	-	-	-	-
	ResNet50 (2021) [71]	76.3	86.5	81.08	67.59	66.88	90.99	0.9201
	ResNet50-Oversample (2021) [71]	72.2	44.3	54.90	61.01	60.39	89.88	0.9
	LSTM (2020) [72]	50	71.7	58.91	49.55	49.04	87.33	0.83
	FCTCNN	84.91	73.44	78.76	67.31	66.87	91.76	0.9254
Dos	KNN-GAN (2022) [2]	-	56.76	63.18	-	-	-	-
	ResNet50 (2021) [71]	100	0	0	0	0	0	0
	ResNet50-Oversample (2021) [71]	32.3	42.9	36.85	0	0	53.42	0.5
	LSTM (2020) [72]	0	0	0	0	0	0	0
	FCTCNN	79.59	53.66	64.10	44.63	42.40	87.90	0.7965
Worms	KNN-GAN (2022) [2]	-	38.46	49.26	-	-	-	-
	ResNet50 (2021) [71]	0	0	0	0	0	0	0
	ResNet50-Oversample (2021) [71]	2.4	84.1	4.66	0	0	0	0
	LSTM (2020) [72]	0	0	0	0	0	0	0
	FCTCNN	100	100	100	100	100	100	1
Generic	KNN-GAN (2022) [2]	-	99.64	98.65	-	-	-	-
	ResNet50 (2021) [71]	98	95	96.47	93.93	93.71	99.1	0.9892
	ResNet50-Oversample (2021) [71]	99.3	93.1	96.10	93.91	93.69	99.03	0.9891
	LSTM (2020) [72]	97.5	92.3	94.82	92.92	92.10	98.88	0.9801
	FCTCNN	98.76	93.61	96.12	93.94	93.93	99.40	0.9907

**Table 4:** Performance comparison of deep models on the CIC-IDS2017 dataset.

Attack Name	Model	Precision	Recall	F1-Score	MCC	Kappa	Specificity	AUC
Normal	Lightweight-ensemble 2023 [63]	99.57	99.85	99.71	99.48	99.48	99.70	0.9991
	FCTCNN	99.61	99.80	99.71	99.50	99.50	99.73	0.9999
DDos	Lightweight-ensemble 2023 [63]	78.92	48.47	60.05	67.55	67.54	95.41	0.981
	FCTCNN	99.99	100	99.99	99.98	99.98	99.99	1
FTP-Patator	Lightweight-ensemble 2023 [63]	0	0	0	0	0	0	0
	FCTCNN	99.99	99.81	99.87	99.74	99.74	99.99	0.999
Port-Scan	Lightweight-ensemble 2023 [63]	99.59	99.52	99.56	99.56	99.56	99.51	0.99
	FCTCNN	99.99	99.99	99.99	9.99	99.99	99.99	0.999
SSH-Patator	Lightweight-ensemble 2023 [63]	0	0	0	0	0	0	0
	FCTCNN	100	99.93	99.96	99.93	99.93	100	0.9997
Brute-Force	Lightweight-ensemble 2023 [63]	94.74	94.36	94.55	49.29	49.29	31.23	0.9361
	FCTCNN	99.32	100	99.65	57.53	49.74	33.33	0.9474
SQL-Injection	Lightweight-ensemble 2023 [63]	99.81	99.81	99.81	57.57	51.69	45.31	0.9585
	FCTCNN	100	99.32	99.65	57.53	49.74	33.33	0.9474

**Table 5:** Results of the proposed model in 8 types of attacks in the TON-IIOT dataset, Windows 10 section.

Attack Name	Precision	Recall	F1-Score	MCC	Kappa	Specificity	AUC
NORMAL	99.99	99.86	99.91	99.80	99.80	99.94	0.9999
DDOS	99.92	099.92	99.92	99.81	99.81	99.89	1
Password	99.83	99.83	99.82	99.68	99.68	99.86	0.9999
XSS	99.66	100	99.83	99.64	99.64	99.62	1
Injection	98.96	98.45	98.71	96.40	96.40	98.14	0.9998
DOS	90.6	98.71	94.47	90.74	90.50	93.10	0.9940
MITM	100	100	100	100	100	100	1
Scanning	100	100	100	100	100	100	1

**Table 6:** Results of the proposed model in 8 types of attacks in the BoT-IoT dataset.

Attack Name	Precision	Recall	F1-Score	MCC	Kappa	Specificity	AUC
Normal	100	100	100	100	100	100	0.9996
UDP	99.99	99.99	99.99	99.99	99.99	99.99	0.9999
TCP	99.95	99.65	99.80	99.79	99.79	99.99	0.9999
Service Scan	91.97	65.05	76.20	72.53	70.92	98.40	0.9486
OS Fingerprint	99.79	99.6	99.69	99.65	99.65	99.97	0.9954
HTTP	99.58	99.79	99.68	92.40	92.37	90.47	0.9971
Data Exfiltration	50	50	50	44.73	44.73	50	0.52
Keylogging	94.73	94.73	94.73	90.74	90.74	94.73	0.978

**Table 7:** Results of the proposed model in 9 types of attacks in the NBaIoT dataset.

Attack Name	Precision	Recall	F1-Score	MCC	Kappa	Specificity	AUC
Normal	99.97	99.98	99.98	99.76	99.76	99.69	0.9998
Mir-Syn	99.99	100	99.99	99.99	99.99	99.99	0.99999
Gaf-Udp	99.96	76.66	86.77	64.09	58.27	99.90	0.8827
Mir-Ack	999.99	99.99	99.99	99.98	99.98	99.98	0.9999
Gaf-Tcp	99.96	99.99	99.97	99.93	99.93	99.91	0.9999
Gaf-Udpplain	100	100	100	100	100	100	0.9999
Gaf-Combo	99.99	97.60	98.73	97.53	97.50	99.90	0.9992
Gaf-Junk	99.94	99.93	99.94	99.88	99.88	99.95	0.9999
Mir-Scan	99.94	99.93	99.94	99.88	99.88	99.95	0.9999

**Table 8:** Comparison of average time (s) across five datasets.

Model/Dataset	Unsw-NB2015	CIC-IDS2017	N_BaIOT_2018	BoT-IOT	TON_IIOT
FCTCNN	0.162	1.113	2.404	2.266	0.124
LSTM (2023) [64]	1.9	1.42	2.63	2.93	0.313
ANN (2023) [64]	0.188	1.23	2.39	2.61	0.298
LSTM (2020) [72]	0.198	1.48	2.71	2.91	0.327
BiLSTM (2020) [72]	0.201	1.53	2.77	2.81	0.351
Lightweight-ensemble 2023 [63]	0.28	1.41	2.38	2.26	0.127
KNN-GAN (2022) [2]	1.33	2.42	2.98	3.12	0.571
ResNet50 (2021) [71]	0.321	0.121	2.48	2.31	0.233

## Data Availability

These data were derived from the following resources available in the public domain: CIC-IDS2017: https://www.unb.ca/cic/datasets/ids-2017.html, accessed on 24 May 2024, BoT-IoT, TON_IoT and UNSW-NB15: https://research.unsw.edu.au/projects/bot-iot-dataset, accessed on 24 May 2024, NBAIOT: https://www.kaggle.com/datasets/mkashifn/nbaiot-dataset, accessed on 24 May 2024.

## Abbreviations

The following abbreviations are used in this manuscript:

Acronyms	Definitions
CNN	Convolutional neural network
IIoT	Industrial internet of things
IDS	Intrusion detection system
FCTCNN	Focal causal temporal convolutional neural network
DoS	Denial of services
DDoS	Distributed denial of services
NIDS	Network-based intrusion detection system
ROC	Receiver operating characteristic
MitM	Man in the middle
